# An updated overview of spectrum of gluten-related disorders: clinical and diagnostic aspects

**DOI:** 10.1186/s12876-020-01390-0

**Published:** 2020-08-06

**Authors:** Nazanin Taraghikhah, Sara Ashtari, Nastaran Asri, Bijan Shahbazkhani, David Al-Dulaimi, Mohammad Rostami-Nejad, Mostafa Rezaei-Tavirani, Mohammad Reza Razzaghi, Mohammad Reza Zali

**Affiliations:** 1 Basic and Molecular Epidemiology of Gastrointestinal Disorders Research Center, Research Institute for Gastroenterology and Liver Diseases, Shahid Beheshti University of Medical Sciences, Tehran, Iran; 2 Gastroenterology and Liver Diseases Research Center, Research Institute for Gastroenterology and Liver Diseases, Shahid Beheshti University of Medical Sciences, Tehran, Iran; 3 Division of Gastroenterology and Liver Diseases, Imam Khomeini Hospital Complex, Tehran University of Medical Sciences, Tehran, Iran; 4 Department of Gastroenterology, South Warwickshire Foundation Trust, Warwickshire, UK; 5 Proteomics Research Center, Faculty of Paramedical Sciences, Shahid Beheshti University of Medical Sciences, Tehran, Iran; 6 Laser Application in Medical Sciences Research Center, Shahid Beheshti University of Medical Sciences, Tehran, Iran

**Keywords:** Gluten, Celiac disease, Diet, gluten-free, Ataxia, Hypersensitivity, Diagnosis

## Abstract

The incidence of gluten-related disorders (GRDs) continues to increase and its global prevalence is estimated at approximately 5% of the population. Celiac disease (CD), dermatitis herpetiformis (DH), gluten ataxia (GA), wheat allergy (WA), and non-celiac gluten sensitivity (NCGS) are the five major GRDs that present with a wide range of clinical manifestations. The diagnosis of GRDs can be challenging because the typical and atypical clinical manifestations of the GRDs overlap. In this review, the current definitions of gluten-related disorders, focusing on their clinical features, diagnostic and therapeutic approaches are presented. We concluded that GRDs are usually diagnosed using a combination of clinical features, serological tests, and histopathological findings. Treatment usually involves dietary modification.

## Background

Gluten-related disorders (GRDs) refer to a group of conditions that are known to be caused by the ingestion of the gluten proteins present in wheat, barley, and rye. GRDs are heterogeneous, reflecting their autoimmune, allergic, and non-autoimmune-allergic etiology. Celiac disease (CD), dermatitis herpetiformis (DH), and gluten ataxia (GA) are considered to be autoimmune. Wheat allergy (WA) and non-celiac gluten sensitivity (NCGS) are considered to be allergic and non-autoimmune-allergic diseases [1–3].

GRDs are estimated to have a global prevalence of approximately 5% [4]. Until two decades ago, CD and other GRDs were considered to be almost exclusively found in European populations. Advances in the development of sensitive and specific serological tests have led to an increase in the diagnosis of GRDs and recognition that these conditions are a significant global health issue [5]. The cultivation of ancient grasses, such as the progenitors of modern wheat and barley, first started in the Fertile Crescent of the Middle East approximately 10,000 years ago. Cultivation of these ancient grasses slowly spread across northern Europe which coincided with the growth of the earliest civilizations and since then symptoms in keeping with GRDs were reported [6–9]. Much later the mechanization of agriculture and most recently, the industrial use of pesticides, nitrogen-based fertilizers, and genetic modification have led to the production of a vast amount of wheat, including new types of wheat with high gluten content. These gluten-rich wheats are used in the global food industry. These rapid changes in the amount and type of wheat being consumed may be responsible for the global increase in the prevalence of GRDs [5, 10]. In a short period of time, in evolutionary timescales, wheat has become one of the most important food sources in the world [1, 6]. Furthermore, the use of ingredients such as Baker’s yeast, instead of natural sourdough, reduces the degradation of immunodominant gluten peptides. This change in cooking techniques, combined with the high gluten wheat, can be another factor responsible for the increasing prevalence of GRDs in recent years [5, 8].

Among the GRDs, CD and DH have been extensively studied and the role of gluten in their pathogenesis has been clearly identified. CD can present with both intestinal and extra-intestinal symptoms including bloating, abdominal discomfort, and fatigue. However, DH typically presents with extra-intestinal symptoms, such as a blistering rash [11]. Patients affected by NCGS also report a wide range of intestinal and extra-intestinal symptoms related to the ingestion of gluten, such as abdominal pain, but the etiology of this condition is less clearly understood than the etiology of CD and DH. The NCGS pathogenesis is completely different from CD [5, 12]. Moreover, WA presents with typical allergy symptoms including rhinitis, eczema, and wheezing caused by the activity of IgE antibodies against gluten and other proteins contained in wheat. The IgE up-regulation may cause transient gastrointestinal presentations including nausea and bloating [4, 5, 12]. Although different GRDs have specific pathophysiological responses to the ingestion of gluten, the same clinical manifestations can make their differential diagnosis challenging [13]. Understanding the clinical presentations and etiology of the GRDs helps clinicians decide upon appropriate investigation and treatment. The present review considers the spectrum of gluten-related disorders, focusing on clinical features, investigations, diagnostic criteria and therapeutic approaches for each of the conditions.

### Celiac disease (CD)

Celiac disease (CD) is a common GRD in which genetic and environmental factors as well as gluten intolerance are the main causes of innate and adaptive immune responses [14–18]. CD is characterized by small intestine mucosal lesions, subtotal, or total intestinal villi atrophy and nutrient malabsorption [19]. The global prevalence of CD is estimated at 1–2% in the general population and 0.3–2.9% in children [20, 21].

CD can be associated with a wide spectrum of manifestations, including intestinal and extra-intestinal symptoms or it can even be asymptomatic [7]. Common intestinal features include chronic and persistent diarrhea, malabsorption, abdominal pain, weight loss, and steatorrhea. Atypical and extra-intestinal manifestations include hepatic hypofunction, iron deficiency anemia, hair loss, osteoporosis, growth retardation, epilepsy, psychiatric disorders, mouth ulcers, muscle weakness, fatigue, arthropathy, delayed onset of puberty in children and infertility in adults [7, 22–25].

Conditions associated with CD include genetic disorders such as Downs syndrome, Turners syndrome and Williams syndrome; autoimmune disorders including type 1 diabetes mellitus (DM1), inflammatory bowel disease (IBD), autoimmune thyroid disorders, autoimmune hepatitis; neurological disorders like ataxia and epilepsy [26, 27].

CD can present at any age after the introduction of gluten to the diet [28]. Children under 2 years old typically present with gastrointestinal symptoms and failure to thrive. Older children and adults typically present with symptoms that are mostly nonspecific and atypical [29–31]. The differences in the clinical presentation of CD may be due to immunological factors, the age of onset, the duration and the extent of disease, degree of small intestinal mucosal inflammation, gender, and family history [26].

**Fig. 1:**
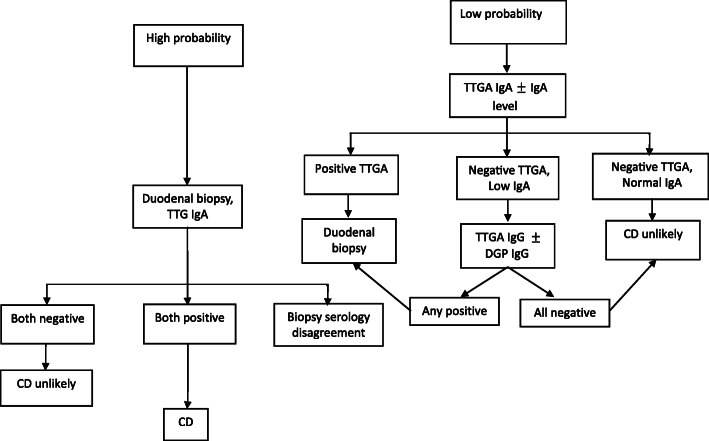
Summary of Rubio Tapia et al. approach of celiac disease

**Fig. 2:**
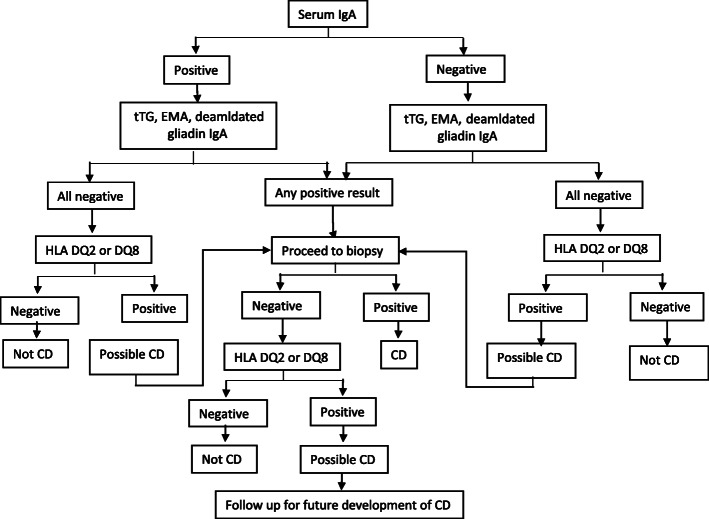
Celiac disease diagnostic testing algorithm adopted from Mayo Medical Laboratories

The diagnosis of CD is challenging and it should be considered when patients present with either intestinal or extra-intestinal symptoms, such as bloating or fatigue. CD is more common in patients with a family history of CD and DM1 than in the general population, even in the absence of gastrointestinal symptoms [32]. The correct diagnosis of CD requires a combination of clinical, serological, and histopathological evaluations [33]. It is recommended that patients with a clinical presentation of CD should undergo serological tests [34]. Several antibodies can be used in CD detection such as anti-tissue transglutaminase (Anti-tTG), anti-endomysial (EMA), and anti-deamidated gliadin peptides (Anti-DGP), IgA and IgG antibodies [32, 35]. Anti-tTG antibodies are the most common serologic markers for CD diagnosis that have 96–98% sensitivity and 88–100% specificity [36, 37]. IgA-tTG is the recommended serological test for the detection of CD. As IgA-deficiency affects 2–3% of CD patients and leads to false-negative results, total IgA levels also need to be measured. In the presence of IgA-deficiency, IgG antibody-based tests (IgG-tTG and/or IgG-DGP) should be used [32, 37]. High tTG-antibody levels (> 5 times the upper normal unit (ULN)) is suggestive of a diagnosis of CD. IgA-EMA antibody-based tests have a high sensitivity and specificity for the diagnosis of CD. These tests can be used as additional and confirmatory serological tests for the initial diagnosis of celiac disease, in conjunction with the measurement of anti-tTG antibodies. Unfortunately, IgA-EMA antibody tests are expensive and user-dependent [37, 38]. An antibody test followed by a small intestinal biopsy evaluation is the gold standard for the definitive diagnosis of CD [36, 39]. Adequate small intestinal sampling is essential in this regard and studies have concluded that at least one biopsy from the duodenal bulb and at least four biopsies from the distal duodenum are needed for an accurate diagnosis [40, 41]. Biopsy appearances in keeping with CD include scalloping, villous flattening, and fissuring of mucosal folds. Small intestinal appearances can become more pronounced with disease progression [17, 40, 41]. According to the Marsh classification, the intestinal biopsy changes are classified on the extent of increased intraepithelial lymphocytes, crypt hyperplasia, and villous atrophy [42]. Corazza and Oberhuberhave also proposed modifications to the Marsh classification [43, 44] (Table 1), and these proposed modifications have been challenged, Marsh et al. [45]. CD diagnostic tests (serologic and endoscopic tests) should be performed when the patient is on a gluten-containing diet to avoid false-negative results [46]. Human leukocyte antigens (HLA-DQ2/DQ8) are the most important genetic risk factors for celiac disease [47]. HLA typing can be used when the results of the serological and histopathological tests are inconclusive and the diagnosis of CD is uncertain [48, 49]. As almost all patients with CD have HLA-DQ2 or HLA-DQ8, the absence of these HLAs makes the diagnosis of CD very unlikely [47, 50, 51]. HLA-DQ2 is found in 90–95% of CD patients, and the other 5% have HLA-DQ8 variant [52]. The European Society for Pediatric Gastroenterology, Hepatology, and Nutrition (ESPGHAN) guidelines suggest that the diagnosis of CD in pediatrics can be made without biopsy evaluation, if the patient has a high level of anti-tTG antibodies (> 10 times ULN) along with testing positive for EMA-antibodies and HLA-DQ2/DQ8 haplotypes. Biopsy evaluation is advised as being essential if either the EMA or HLA results do not support a diagnosis of CD. The guidance also suggests that if patients have symptoms suggestive of CD, but their anti-tTG level is < 10-time ULN, or when the patients are asymptomatic, but the anti-tTG level is > 10-time ULN, endoscopic intestinal biopsy is advisable [53]. In addition to the ESPGHAN guidelines, Rubio et al. [32] (Fig. 1) and Mayo medical laboratories have given an algorithm for the diagnosis of CD (Fig. 2) [7]. There are emerging methods for the diagnosis of CD. These methods include video capsule endoscopy (VCE), biochemical tests such as measurement of intestinal fatty acid-binding protein (I-FABP), radiology methods and intestinal permeability tests that can provide additional information about the appearance and function of the small intestinal mucosa, increasing the detection and evaluation of CD [54].

**Table 1:** Histological classifications commonly used for celiac disease

	HISTOLOGICAL CRITERIA		MARSH MODIFIED (OBERHUBER)	CORAZZA
Increased intraepithelial lymphocytes (> 40% for Marsh, > 25% for Corazza)	Crypt hyperplasia	Villous atrophy		
No	No	No	Type 0	None
Yes	No	No	Type 1	Grade A
Yes	Yes	No	Type 2	
Yes	Yes	Yes (partial)	Type 3a	Grade B1
Yes	Yes	Yes (subtotal)	Type 3b	
Yes	Yes	Yes (total)	Type 3c	Grade B2

#### Video capsule endoscopy (VCE)

Video capsule endoscopy (VCE) is a non-invasive procedure that provides high-resolution images of the entire small bowel mucosa [54–58]. Chang et al. [55] in their meta-analysis study showed that VCE is sensitive (89%) and specific (95%) for CD detection [55].

#### Intestinal fatty acid-binding protein (I-FABP) evaluation

Intestinal fatty acid-binding protein (I-FABP) is a water-soluble protein predominantly expressed in the small intestine epithelial cells. When enterocytes are damaged, I-FABP is released into the systemic circulation. Serum I-FABP has the theoretical potential to be a non-specific marker of small bowel inflammation in conditions such as CD [54, 59, 60].

#### Radiology methods

Radiological methods are routinely used to visualize the small bowel, but not routinely used for the diagnosis of CD [54, 61]. Advanced imaging techniques including computed tomography (CT) and magnetic resonance (MR) modalities are frequently used for the evaluation of small-bowel diseases. CT and MR abnormalities have been reported in CD [54, 61–63].

#### Intestinal permeability tests

Permeability tests (e.g., D-xylose test, sucrose, lactulose-mannitol ratio) can be used to measure the small bowel permeability which is increased in CD. Abnormalities in the small intestine are not specific for CD. Permeability tests have low sensitivity (65%) and low specificity (74%) for the diagnosis of CD [32, 54, 64].

#### Saliva tests

Furthermore, saliva tests, measuring tTG, are being evaluated. However, currently, there is insufficient evidence to evaluate the benefit to patients by employing these tests and the sensitivity of saliva tests in being able to diagnose CD [65, 66].

The cornerstone of the treatment for CD is a lifelong gluten-free diet (GFD) and no other strategies are comparable to a GFD in treating this disorder [7, 15, 26].

### Dermatitis herpetiformis (DH)

Dermatitis Herpetiformis (DH) is a chronic, autoimmune, and recurrent cutaneous-intestinal disorder identified in genetically susceptible individuals, which is often associated with CD [67, 68]. Anti-tTG antibodies that are produced in response to gluten exposure can also recognize epidermal transglutaminase (ETG). ETG is structurally homologous to tTG and is the main antigen in DH [67]. Deposition of IgA antibodies in the superficial papillary dermis of DH patients causes vesiculobullous, pruritic, and localized lesions. DH affects the extensor surfaces such as elbows, buttocks, knees, and scapular areas [67, 69]. DH is prevalent in Scandinavian countries and the UK and typically presents in patients aged between 15 and 40 years. Males are affected more than females, with a ratio of 3:2, but interestingly it is more prevalent in females than males under the age of 20 years [67, 70].

DH patients rarely present gastrointestinal symptoms, although all of them have gluten sensitivity. Approximately two-thirds of patients have some degree of villous atrophy, and one third has intraepithelial lymphocytosis [67].

DH is associated with a wide range of autoimmune diseases, such as T1DM, pernicious anemia, Addison’s disease, vitiligo, alopecia areata, and rheumatoid arthritis, systemic lupus erythematosus, Sjogren ‘s syndrome and thyroid abnormalities [71].

Skin biopsy evaluation is advised in patients with clinical manifestations that are suggestive of DH and should be taken close to, but not from any vesicles. Direct immunofluorescence (DIF) should be performed on skin biopsy specimens [72]. The classic histopathological finding of DH is a sub-epidermal cleft rich in neutrophils and eosinophils that presents at the dermal papillae [67, 72, 73]. For patients with clinical presentations of DH, but negative DIF, other confirmatory tests (such as anti-tTG antibody level) can be applied. Both CD and DH patients have raised tissue transglutaminase specific auto-antibodies level in serum and small bowel mucosa [69, 74]. An additional serological test used in the diagnosis of DH is the measurement of serum EMA-antibody levels; this has sensitivity and specificity of at least 90% [67, 75]. Recently, some researchers have introduced the anti-deamidated gliadin peptide antibody (anti-DPG) as a possible marker of DH. Anti-DPG has a high sensitivity (between 84 and 90%), and it can be useful in the diagnosis of cases with suspected DH with negative anti-tTG [76, 77]. ETG is the key auto-antigen in DH, therefore, it can efficiently differentiate DH from other dermatological diseases. Its sensitivity and specificity have been reported between 52 and 90%, and 93 and 100% respectively, nevertheless ETG is currently not approved as a diagnostic marker of DH [73, 78–80]..

DH is treated with a GFD combined with pharmacological treatment including sulfones, such as dapsone, and sulfonamides [81]. Dapsone is an anti-inflammatory agent that downregulates neutrophil chemotaxis, reduces the release of leukotrienes and prostaglandins, and thus prevents tissue damage [82]. Possible side effects of dapsone include hematological disturbances such as methemoglobinemia and agranulocytosis [83].

### Gluten ataxia (GA)

Gluten ataxia (GA) is a type of cerebellar ataxia caused by exposure to gluten in sensitive and genetically susceptible individuals [84]. GA is an autoimmune disorder characterized by the presence of a cerebellar injury, affecting mainly Purkinje cells. In most cases of GA, there has been a previous diagnosis of CD or NCGS with digestive symptoms [84]. Several studies have suggested possible mechanisms for the development of GA in CD. Impaired intestinal absorption leading to vitamin E deficiency can cause spinocerebellar degeneration [85]. Malabsorption can also cause damage to the serotonin-containing neurons in the cerebellum, and brainstem [86]. Immunological and inflammatory processes may also be important in the etiology of GA. There is a cross-reactivity between antigenic epitopes located at the level of Purkinje cells and gluten-related antibodies. In susceptible individuals, anti-gliadin antibodies may have a clinically significant direct or indirect neurotoxic effect [87].. Hadjivassiliou et al. [84] estimated that GA accounts for approximately 15% of all ataxias and 40% of all idiopathic sporadic cerebellar ataxias. GA is more common in the USA and Europe than in Asia. It typically affects males and females aged over 50 years [88].

The clinical manifestations of GA are similar to those of other ataxias and include ocular signs like gaze-evoked nystagmus (84%), dysarthria (66%), upper limb ataxia (75%), lower limb ataxia (90%), gait ataxia (100%) and additional movement disorders such as myoclonus, chorea, palatal tremor and opsoclonus myoclonus [89]. GA is characterized by gradual onset of gait ataxia, associated with peripheral neuropathy. Occasionally, it can be rapidly progressive, similar to paraneoplastic cerebellar degeneration [90]. The absence of autonomic dysfunction helps differentiate these patients from patients with the cerebellar type of multiple system atrophy (MSA-C) [91].

The diagnosis of GA is supported by the presence of anti-gliadin, anti-tTG, and anti-TG6^1^ (when available) antibodies in the serum. The optimum diagnostic strategy for patients with suspected GA remains uncertain. Published studies have suggested that the IgA anti-gliadin antibody is more specific than the IgG anti-gliadin antibody test [92–97], but Hadjivassiliou et al. [89] reported that IgG anti-gliadin antibody is a better marker of gluten ataxia, because of its high sensitivity. Since the level of anti-gliadin antibodies is 5–12% in the general population, some clinicians believe that anti-gliadin antibody tests cannot be used for the diagnosis of GA [89]. Studies of GA patients have shown that anti-tTG antibodies are present in the brain, supporting a possible pathogenic role in the etiology of the condition. If CD serology is positive, then obtaining intestinal biopsies to look for evidence of CD should be considered [96]. Magnetic resonance imaging (MRI) can also be used for GA diagnosis. MRI studies of GA patients show the presence of moderate cerebellar atrophy in up to 60% of patients [87].

GA patients should be treated with a strict GFD. In addition, studies have shown that immunotherapy (steroid, intravenous immunoglobulins (IVIG)) can be an effective treatment for such patients [98]. As GA is a progressive disorder in which neurons and Purkinje cells are destroyed over time, the response to treatment depends on the time interval between the onset of the GA and treatment [99, 100].

### Wheat allergy (WA)

Wheat allergy (WA) is one of the most common food allergies (since wheat provides 70% of dietary proteins), and should be considered as a serious health problem worldwide [101]. In contrast to CD, different wheat components such as water-soluble (albumin and globulin) and water-insoluble (glutenin and gliadin) proteins contribute to the development of wheat allergy [102–104]. WA is more common in pediatric practice than adult medicine (the mean age of onset for WA is 5.5 years (3–16 years)) and the global prevalence of WA is reported at 0.5–1% [105–108]. Wheat allergy as a subgroup of food hypersensitivity is categorized into two groups; IgE-Mediated and non-IgE-Mediated WA [108–110].

#### IgE-mediated WA

Allergen ingestion (food allergy), inhalation (respiratory allergy), or skin contact (dermal allergy) causes T helper type 2 activation and immunoglobulin E (IgE) production by B and T cells [108]. Cross-linking of IgE with gluten peptides triggers the release of chemical mediators such as histamine from basophils and mast cells, leading to the clinical manifestations of allergic responses, including WA [109, 111]. The most common manifestations of WA due to these mechanisms include gastrointestinal (abdominal pain, nausea, vomiting, diarrhea, bloating), dermal (itching, eczema, pruritus, dizziness, atopic dermatitis, swelling, redness), respiratory (rhinitis, asthma, sneezing, chronic cough), circulatory (flushing, angioedema), cerebral (disturbed or foggy thinking, headache, dizziness, migraines) symptoms [108, 110]. These manifestations can be immediate (minutes to an hour after ingestion) or delayed (a few hours after oral ingestion of wheat) [112]. Additionally, manifestations can be mild or life-threatening according to the severity of the reaction [108, 110].

Wheat-dependent exercise-induced anaphylaxis (WDEIA) is a particular type of IgE-mediated WA. Allergic reactions occur when ingestion of wheat products is accompanied by triggering cofactors such as exercise (within 1–3 h). Symptoms of WDEIA include pruritus, angioedema, flushing, dyspnea, dysphagia, chest pain, syncope, headache, nausea, diarrhea, and hoarseness [3, 113, 114].

#### Non-IgE-mediated WA

Non-IgE-mediated wheat allergy (delayed onset wheat allergy) is closely associated with eosinophilic esophagitis (EOE) or eosinophilic gastritis (EG) [108]. This type of wheat allergy has different intestinal and extra-intestinal symptoms, including indigestion, vomiting, diarrhea, headaches, and arthralgia that are delayed for several hours or even days after ingestion of allergens. It is associated with other food allergies (e.g., milk, egg white, peanuts) [115, 116].

The diagnosis of a WA is dependent on clinical suspicion and a detailed dietary history. Diagnosis of a WA is supported by skin patch testing (SPT) and measurement of total IgE and wheat specific IgE [1, 108, 117]. Although these tests are sensitive (73% for SPT, 83% for specific IgE), they do not have enough specificity (73% for SPT, 43% for specific IgE) to establish a diagnosis of WA [1, 118]. Furthermore, the specific IgE level is not related to the severity of symptoms and its diagnostic level varies depending on the type of WA [108, 119]. Serum IgE assays for Tri a 19 (omega-5-gliadin) and Tri a 36 (low molecular weight glutenin) have been introduced into pediatric practice to support the diagnosis of WA. The assays are useful for diagnosing infants (< 1-year age) with suspected WA [120, 121]. Flow cytometric basophil activation test (BAT) has been applied in research settings to evaluate the allergen-induced activation of basophils, which quantify the basophils response to specific allergens. The BAT has a high specificity and sensitivity for the diagnosis of WA, but requires specialized laboratory equipment [122, 123]. To confirm the diagnosis of WA an open food challenge (OFC) and bronchial challenge tests (BCT) are sometimes considered [108]. In OFC, increasing doses of wheat are administered at 30-min intervals. Numerous investigations have reported that wheat OFC is safe with 30–50% failure; however, it can be associated with fatal reactions and anaphylaxis [108, 124]. The BCT is the gold standard to confirm the diagnosis of occupational respiratory diseases; 30 mg of placebo flour is sniffed into one nostril, and after 10 min, the same procedure is repeated in another nostril, it can be repeated up to 3 times. Patients with a WA may develop itching, sneezing, rhinitis, and a decrease in FEV1 during the BCT [108, 124]. Finally, a double-blind placebo-controlled food challenge (DBPCFC) is considered to be the ‘gold standard’ diagnostic approach for WA. As the DBPCFC is time-consuming, expensive and resource-intensive with the potential to induce anaphylaxis, it is rarely used in standard practice [124–126]. IgG / IgG4 antibodies have an important role in inducing natural dietary tolerance and their serum level increases in the setting of resolving food allergy. Increased allergen-specific IgG levels have not been validated for use in WA, because levels of allergen-specific IgG levels can be elevated in other gastrointestinal inflammatory diseases such as CD. Furthermore, increased IgG levels can be observed in more than half of the general population, as a response to most common food elements [127, 128].

WA can be transient and its symptoms may improve or disappear within a few years of wheat withdrawal especially in children, but it can persist in adults as a lifelong disorder [129]. High levels of WA specific IgE antibodies for several years, despite the elimination in diet, indicate persistent WA [106]. Patients with WA should be educated on allergen avoidance and get nutritional support from dieticians. The only treatment approach for these patients is an adherence to a wheat-free diet; nevertheless, they can consume gluten from non-wheat sources. Epinephrine must be immediately administered in the case of wheat exposure and severe reactions [108, 130, 131].

### Non-celiac gluten sensitivity (NCGS)

Non-celiac gluten sensitivity (NCGS) refers to a reaction to gluten leading to intestinal and extra-intestinal manifestations that are not mediated by an allergic or immunologic response [132, 133]. The terms gluten sensitivity, gluten hypersensitivity, and non-celiac gluten intolerance also refer to this condition [133–135]. NCGS is more common in adult females (F/M 6:1) and its prevalence is estimated at 0.6–13% of the general population [103, 136, 137].

NCGS can cause a wide variety of symptoms including abdominal pain, diarrhea, weight loss, headache, fatigue, malaise, muscle pain, recurrent oral ulceration, and depression [138–140]. Recent studies proposed that besides gluten, other components of wheat, such as poorly fermentable, poorly absorbed, short-chain carbohydrates, and wheat amylase-trypsin inhibitors may contribute to the development of NCGS [136, 137, 141–143]. NCGS symptoms occur in a few hours or days after gluten ingestion, that resolve on a GFD and relapse after a gluten challenge [144, 145]. A significant proportion of NCGS patients are self-diagnosed and start a GFD without medial consultation [146, 147].

Currently, the diagnosis of NCGS is dependent upon a clinical assessment of symptoms and exclusion of WA and CD – the “Salerno Experts’ Criteria” (the probability of CD and WA must be ruled out) in patients on diets that contain gluten [146]. In other words, NCGS should be considered in patients with negative WA and CD tests (the small intestine of NCGS patients is usually normal, and serum tTG-antibodies and EMA-antibodies are negative) [148, 149]. HLA-DQ2/DQ8 haplotypes are found in approximately 50% of NCGS patients. These haplotypes are not required for the condition to develop, and HLA typing cannot be used to confirm or exclude a diagnosis of NCGS [1, 138]. As there is no specific NCGS diagnostic biomarker and test, a therapeutic trial of a GFD may be considered. Following a diagnosis of NGCS, patients may be asked to undergo a double-blind, placebo-controlled (DBPC) gluten challenge [140, 150]. Patients diagnosed with NCGS showed a significant worsening of symptoms after gluten consumption [151]. However, this exclusion diagnostic protocol remains cumbersome and is not easy to perform in daily clinical practice. There is an increasing need for having a clear diagnostic process that will lead to a definitive diagnosis in suspected NCGS individuals [144, 152]. To date, in various studies, the attempt has been made to identify the predictive pattern of NCGS. In the absence of a definitive test to diagnose NCGS, studies continue to focus on serum markers of wheat intolerance. Studies have shown that IgG anti-gliadin antibodies (IgG-AGA) are present in approximately 56% of NCGS cases and over 80% of CD cases, compared to 2–8% of the general population [146, 153, 154]. Duodenal biopsies from patients with NCGS are typically reported as normal, but detailed analysis suggests a mild increase in intraepithelial lymphocytes, increased expression of claudin 4, Toll-like receptor 2 (TLR2) and interferon-gamma (IFNγ) and increased goblet cell number in this condition [140, 155].

There are conflicting data on intestinal permeability in NCGS [156, 157]. In the primary study conducted by Sapone et al. [158] in 2011, gut permeability of CD and NCGS patients was determined using the urine lactulose/mannitol (LA/MA) test. The results of this study demonstrated a significantly lower small intestinal permeability in NCGS compared to CD patients and controls. The study also reported a high expression of claudin-4 mRNA, in duodenal biopsies from NCGS patients compared to the other patient groups, consistent with the finding of decreased intestinal permeability [158]. Hollon et al. [159] in their ex vivo study in 2015, evaluated changes in transepithelial electrical resistance (TEER) of tissue biopsies from active CD patients (ACD), CD patients in remission, patients with non-celiac gluten sensitivity and controls exposed to pepsin-trypsin digested gliadin (PT-gliadin). The results of their study demonstrated that gliadin exposure reduced TEER and increased intestinal permeability in all patient groups compared to controls. These results suggest that NCGS patients have abnormal intestinal permeability [159]. The study conducted by Uhde et al. [155] on individuals with wheat sensitivity in the absence of CD in 2016, also demonstrated the presence of enterocyte injury, increased intestinal permeability, and microbial products translocation in these patients. This study reported that the increased intestinal permeability was accompanied by an increase in the serum levels of the different biomarkers such as soluble CD14, lipopolysaccharide-binding protein (LBP), and fatty acid-binding protein 2 (FABP2). They proposed that these biomarkers, if validated in subsequent analysis, could be useful as possible NCGS diagnostic tools [155]. Furthermore, Barbaro et al. [160] in 2015 showed significantly high zonulin serum levels, a potential biomarker for monitoring changes in intestinal permeability, following gluten exposure. They noted that zonulin can contribute to NCGS pathophysiology and has a correlation with symptoms in NCGS patients [160]. In the past, patients with NCGS were frequently misdiagnosed. These patients were often believed to have an underlying psychiatric disorder [139, 161]. Unlike CD, NCGS is not considered a high risk for long-term complications or nutrient deficiencies. There is no need to screen the relatives of patients with NCGS [143, 162].

The only way to treat the NCGS is GFD adherence [163]**.**

## Conclusions

Gluten is a dietary protein that is widely used in the global food industry. Genetic and environmental factors predispose individuals to a wide range of GRDs. The GRDs are a diverse group of conditions with different etiologies and clinical manifestations that can overlap. Understanding the underlying etiology of the GRDs guides the diagnosis and management of these globally significant and frequently overlooked conditions.

## Supplementary information

**Additional file 1 Table S1**. Summary of clinical and diagnostic aspects of gluten-related disorders.

## Data Availability

Not applicable.

## Abbreviations

Anti-DGP: Anti-deamidated gliadin peptides
Anti-tTG: Anti-tissue transglutaminase
CD: Celiac disease
DBPCFC: Double-blind placebo-controlled food challenge
DH: Dermatitis herpetiformis
DIF: Direct immunofluorescence
DM1: Type 1 diabetes mellitus
EG: Eosinophilic gastritis
EMA: Anti-endomysial
EOE: Eosinophilic esophagitis
ETG: Epidermal transglutaminase
GA: Gluten ataxia
GFD: Gluten-free diet
GRDs: Gluten-related disorders
HLA: Human leukocyte antigens
IBD: Inflammatory bowel disease
Ig: Immunoglobulin
MRI: Magnetic resonance imaging
NCGS: Non-celiac gluten sensitivity
OFC: Open food challenge
SPT: Skin prick test
ULN: Upper normal unit
WA: Wheat allergy
WDEIA: Wheat-dependent exercise-induced anaphylaxis

## Footnotes

1 Anti-transglutaminase 6 Antibody
