# Supplementary material for: An updated overview of spectrum of gluten-related disorders: clinical and diagnostic aspects
Source: BMC Gastroenterol. 2020 Aug 6;20:258. doi: 10.1186/s12876-020-01390-0 (PMC7409416; doi:10.1186/s12876-020-01390-0)
Supplement: Supplementary file 1 — Additional file 1 Table S1. Summary of clinical and diagnostic aspects of gluten-related disorders. [file 12876_2020_1390_MOESM1_ESM.docx]

**Supplement table. Summary of clinical and diagnostic aspects of gluten-related disorders.**

|  | **Incidence** | **Gender** | **Clinical features** | **Associated disorders** | **Serology** | **Genetic** | **Histology** | **Skin tests** | **Treatment** |
| --- | --- | --- | --- | --- | --- | --- | --- | --- | --- |
| **Celiac Disease** | 1-2% worldwide | Female > Male | 1. intestinal symptoms: diarrhea, malabsorption, abdominal pain,  2. extra-intestinal symptoms: iron deficiency, neurological and psychological disorders, growth retardation, …  3. no symptoms. | 1.genetic disorders: down syndrome, turner syndrome, Williams syndrome  2. autoimmune syndromes: DM1, IBS, thyroid disorders, …  3.neurological disorders: ataxia, epilepsy | 1. ANTI-TTG: the most common marker with 95% sensitivity and 95% specificity.  2. ANTI-EMA:  One of the first line tools, but expensive with 96% sensitivity and close to 100% specificity.  3. AGA: it is not recommended because of low sensitivity (80%) and low specificity (80-90%) | HLA-DQ2 is found in 95% of CD patients and HLA-DQ8 is found in the other 5%. | Small intestinal biopsy is a gold standard for CD diagnosis that includes: crypt hyperplasia, flattening of intestinal villi, notching of mucosal folds, decreased enterocyte height, increased intestinal intraepithelial lymphocytes.  This method has a 96% sensitivity. | Not used. | Gluten free diet. |
| **Non-Celiac Gluten Sensitivity** | 0.6-13% | More common in females than males (F/M 6:1) | 1. Intestinal symptoms: abdominal pain, diarrhea, constipation,  2. Extra-intestinal symptoms: malaise, anemia, psychological symptoms. | IBS, other autoimmune disease. | It is diagnosed after ruling out celiac disease and wheat allergy.  DBPC gluten challenge in the diagnostic gold standard.  1. ANTI-AGA: its prevalence is lower than in CD. | HLA-DQ2/DQ8 is found in 50% of NCGS patients. | The intestinal biopsy includes: increased level of TLR-1, 2 receptor, CD3, intraepithelial lymphocytes. | Not used. | Gluten free diet. |
| **Wheat allergy** | 0.5-1% |  | 1.IGE-mediated WA includes: food allergy, respiratory allergy, contact urticaria, WDEIA, anaphylaxis.  2.NON-IgE mediated WA includes: eosinophilic esophagitis, eosinophilic gastritis. | There is an association with allergy to milk, egg, peanut, … | DBPCFC is the diagnostic gold standard.  1.ANTI-IgE: with 83% sensitivity and 43% specificity.  2.ANTI-IGG/IGG4: it is not recommended as a diagnostic test.  3.BAT (flowcytometric basophil activation testing)  4.Tri a 19, Tri a 36: useful in infants and WDEIA. |  |  | Skin prick test (SPT):is performed in the volar surface of forearm using the extract of wheat. A wheal size >_ 3mm within 15min is considered positive. with 73% sensitivity and 73% specificity. | 1. Wheat free diet.  2. EPINEPHRIN administration in case of exposure and anaphylaxis. |
| **Gluten-Ataxia** | Common in the USA and Europe, rare in Asia. | Female=male | A cerebellar ataxia with Ocular signs like gaze-evoked nystagmus (84%), dysarthria (66%), gait ataxia (100%), upper limb ataxia (75%), lower limb ataxia (90%).  Less than 10% of the patients have GI symptoms. |  | 1. ANTI-GLIADIN ANTIBODY: this marker is neurotoxic and high in 12-41% of the patients with ataxia and 5-12% in normal people. THE LEVEL >20 IS POSITIVE. Its specificity ranges between 82-95%.  2. ANTI-DGP.  3. ANTI-TG6: is more specific and sensitive. | There is no need to measure HLA-DQ2/DQ8. | The autopsies of the cerebellum include: patchy loss of Purkinje cells, astrocytic gliosis, vacuolation of neutrophil and diffuse infiltration of T cells. | Not used. | 1.gluten free diet  2.immunotherapy, especially IVIG therapy |
| **Dermatitis herpetiformis** | More prevalent in Scandinavian counties and the UK. | Male> female (3:2), but this ratio is reversed in younger ages. | The grouped vesicobullous lesions on the erythematous or erythematopapulous area on extensor surfaces. / Rarely involves the oral cavity. / Rarely present GI symptoms. | Autoimmune disorders and lymphoma. | 1. ANTI-TTG: with 47-95% sensitivity and >90% specificity.  2. ANTI-ETG: the key autoantibody of DH with 52-90% sensitivity and 92.8-100% specificity.  3. ANTI-EMA: Have 52-100% sensitivity and 100% specificity.  4. ANTI-AGA: its sensitivity is between 84-90% and useful in cases with negative anti-TTG. | HLA-DQ2/DQ8 has a role in the occurrence of DH with a high sensitivity close to 100% and a high negative predictive value. | 2/3 of the patients have some degree of villous atrophy and 1/3 has intraepithelial lymphocytosis. | 1. Direct immunofluorescence of perilesional areas (healthy skin); is the gold standard test and it includes granular IgA deposits along the dermal-epidermal junction and on the tip of the dermal papillae.  2. Histopathological analysis: skin biopsy of erythematous area beside the vesicle that shows sub epidermal cleft with neutrophils and eosinophils in the dermal papillae. | 1. Gluten free diet.  2. medication like dapsone and sulfonamides. |
